# Rheumatic Dysmenorrhea

**Published:** 1903-10

**Authors:** 


### RHEUMATIC DYSMENORRHEA.

| | | |
|---|---|---|
| ℞ | Tr. cimicifugæ | 2 ozs. |
| | Tr. stramonii | ½ oz. |
| | Tongaline, q. s. ad | 4 ozs. |

M. Sig.—A teaspoonful in water at meal times.

